# Method development for on-line species-specific sulfur isotopic analysis by means of capillary electrophoresis/multicollector ICP-mass spectrometry

**DOI:** 10.1007/s00216-020-02781-8

**Published:** 2020-07-01

**Authors:** Sebastian Faßbender, Katerina Rodiouchkina, Frank Vanhaecke, Björn Meermann

**Affiliations:** 1 Federal Institute for Materials Research and Testing (BAM), Division 1.1 - Inorganic Trace Analysis, Richard-Willstätter-Str. 11, 12489 Berlin, Germany; 2 Department of Chemistry, Atomic & Mass Spectrometry – A&MS research unit, Ghent University, Campus Sterre, Krijgslaan 281-S12, 9000 Ghent, Belgium

**Keywords:** River water sulfate, Environmental speciation, Sulfur isotopes, Species-specific isotopic analysis via on-line CE/MC-ICP-MS, Multiple-injection sample-standard bracketing approach

## Abstract

In this work, a method for species-specific isotopic analysis of sulfur via capillary electrophoresis hyphenated on-line with multicollector ICP-MS (CE/MC-ICP-MS) was developed. Correction for the mass bias caused by instrumental mass discrimination was realized via external correction with multiple-injection sample-standard bracketing. By comparing the isotope ratio measurement results obtained using the newly developed on-line CE/MC-ICP-MS method with those obtained via traditional MC-ICP-MS measurement after analyte/matrix separation by anion exchange chromatography for isotopic reference materials and an in-house bracketing standard, the most suitable data evaluation method could be identified. The repeatability for the sulfate-δ^34^S value (calculated from 18 measurements of a standard conducted over seven measurement sessions) was 0.57‰ (2SD) and thereby only twice that obtained with off-line measurements (0.30‰, *n* = 68). As a proof of concept for analysis of samples with a real matrix, the determination of the sulfur isotopic composition of naturally present sulfate was performed for different river systems. The CE/MC-ICP-MS results thus obtained agreed with the corresponding off-line MC-ICP-MS results within the 2SD ranges, and the repeatability of consecutive δ^34^S measurements (*n* = 3) was between 0.3‰ and 1.3‰ (2SD). Finally, the isotopic analysis of two different S-species in a river water sample spiked with 2-pyridinesulfonic acid (PSA) was also accomplished.

**Electronic supplementary material:**

The online version of this article (10.1007/s00216-020-02781-8) contains supplementary material, which is available to authorized users.

**Graphical abstract:**
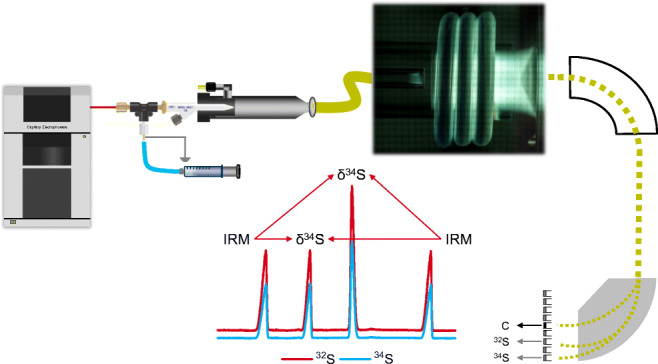
The CE/MC-ICP-MS method developed allows for species-specific S isotopic analysis in samples containing multiple species. Mass bias is corrected for by multiple-injection sample-standard bracketing, while the repeatability (2SD) of the resulting ^34^δ-values is <1‰.

## Introduction

In many scientific fields, isotopic analysis can offer valuable information, e.g., for tracing the origin of minerals, agricultural products of plant or of animal origin or artifacts (provenance determination), for age determination (geochronological dating), the elucidation of reaction mechanisms, and lately also for medical diagnosis [1]. Multicollector inductively coupled plasma-mass spectrometry (MC-ICP-MS) is the technique most often used for high-precision isotopic analysis in such contexts. Most frequently, bulk isotope ratio measurements are performed, thus providing the isotopic composition for the entire elemental content of the sample. However, the analyte is often present in different physical/chemical forms (species) strongly affecting the properties of the element [2]. Separating species of interest from one another (and from matrix components) prior to isotope ratio measurement can provide valuable species-specific isotopic information [3, 4].

Particularly, species-specific isotopic analysis of sulfur (S) has many possible applications. For protein isotopic analysis, S is an element of great interest because it is the only covalently bound constituent of proteins that can be analyzed for its isotopic composition by MC-ICP-MS. In proteins and peptides, S is present in the amino acids cysteine (Cys) and methionine (Met), which are already targets for protein quantification by single-collector ICP-MS [5].

Liver diseases were shown to cause a small (<1‰) yet systematic shift in the bulk S isotopic composition of serum and red blood cells [6] as well as an increased variation in the δ^34^S value in serum and red blood cells of affected patients compared to a control group [7]. In such cases, it is clear that species-specific isotopic analysis could offer more detailed and/or additional information, as it would enable such effects to be related to distinct species. By analyzing only the relevant S species, the measured isotope fractionation would not be blurred by non-fractionating species. Since S is present in all proteins, in peptides and several other important biomolecules, the range of potential applications is virtually unlimited. Therefore, the development of methods capable of species-specific isotopic analysis of S could eventually help deciphering the role of proteins in various physiological and pathological processes.

Outside of the life sciences, S is also a common constituent of environmental contaminants (e.g., sulfur dioxide, pharmaceuticals, biocides) and natural compounds (e.g., sulfate), which can sometimes be traced back to their source by isotopic analysis. By measuring the S isotope ratio in sulfur dioxide and sulfate in precipitation and aerosols, it could be determined whether the sulfur is derived from sea salt or is of biogenic or anthropogenic (combustion of fossil fuels) origin [8–11]. The isotope fractionation of S in the environment is substantial. Species-specific isotopic analysis of sulfate in river water (by precipitation as BaSO_4_ and subsequent analysis with IRMS) has been performed for many different river systems located in Asia, Europe and North America, revealing a large range of δ^34^S values between −4‰ and +18‰ depending on the geological background, anthropogenic influences and season of the year [12–15]. In a review based on data for 13,000 natural samples, a typical range of δ^34^S between −5‰ and +25‰ was reported with extreme values of −55‰ and +135‰ [16].

Up to now, on-line sulfur species-specific isotopic analysis is rarely described in literature, and only applications using gas chromatography (GC) or ion chromatography (IC) hyphenated to MC-ICP-MS have been reported [17–22]. The groups using IC have installed an additional post-column desolvation unit to generate a dry aerosol and mitigate the interference from O_2_^+^ species [18, 22]. The use of desolvation systems was also reported in works conducting sulfur isotopic analysis by non-hyphenated MC-ICP-MS, as water is the most important source of O in the plasma [20, 23–25]. In GC, a gaseous mobile phase is used, and thus a wet aerosol is not an issue; furthermore, a gaseous reference standard (SF_6_) can be applied [17, 19, 20]. However, GC is only suitable for volatile or semi-volatile and thermostable species because derivatization will most likely lead to species transformation and a loss of the pristine species information [2]. Also, chromatography as such can lead to species transformation due to the instability of species caused by interactions with the stationary phase [26–29]. As these interactions may interfere with speciation analysis by chromatography, capillary electrophoresis (CE) can be used as an alternative separation method. It is applicable to a wide range of analytes regarding, e.g., molecular size and polarity, and has many parameters that can be tuned and optimized for a tailored purpose. The small sample volumes needed and short run durations can also be important advantages over chromatographic techniques, especially in the case of biological samples.

However, despite its potential, up to now, CE/MC-ICP-MS has only been used for on-line species-specific isotopic analysis by one group. Martelat et al. developed an approach for isotopic analysis of uranium and plutonium with a spray chamber accommodating two nebulizers, one for introducing the CE flow and the other for the bracketing standard, which was introduced during time segments without electrophoretic peaks [30]. Given the interference issues for sulfur arising from the introduction of liquids, the potential of the approach for isotopic analysis of this element must be thoroughly evaluated.

In this work, a CE/MC-ICP-MS hyphenated setup for species-specific isotopic analysis of sulfur was developed, while a multiple-injection sample-standard-bracketing approach was used to correct for instrumental mass bias. The method was applied to the analysis of isotopic reference materials (IRMs), and the CE/MC-ICP-MS results were compared with those of traditional bulk MC-ICP-MS measurements following off-line anion exchange chromatography for analyte/matrix separation. As a first proof-of-concept application, water samples from different river systems were analyzed for the sulfur isotopic composition of natural sulfate. Based on literature data, the variation in the sulfate-δ^34^S value of river water is substantial, rendering river water sulfate an ideal target species for a proof-of-concept application. Also, the feasibility of on-line isotopic analysis of a degradation product of zinc pyrithione (Zn(PT)_2_), an antifouling biocide — as a potential environmental contaminant — next to sulfate in river water is demonstrated.

## Materials and methods

### Instrumentation

A Mira Mist CE nebulizer (Burgener Research Inc., Mississauga, Canada) was used to hyphenate the Agilent 7100 CE system (Agilent Technologies, Waldbronn, Germany) to a Neptune Plus MC-ICP-MS unit equipped with 10^11^ Ω resistors or an Element 2 sector-field ICP-MS (ICP-SFMS) (both from Thermo Fisher Scientific, Bremen, Germany) for sulfur isotope ratio measurements and quantification, respectively. The sheath liquid was supplied via a 100 Legacy syringe pump (KD Scientific, Holliston, MA, USA) equipped with gastight glass syringes from Hamilton Company (Reno, NV, USA). A drainless 8 mL quartz spray chamber with a make-up gas connection (AHF Analysentechnik, Tübingen, Germany) was used. It was heated by an IR lamp to avoid condensation on the walls and to aid desolvation. The spray chamber was connected to the ICP torch by Tygon tubing. Fused silica capillaries with 75 μm i.d. (Polymicro Technologies, Phoenix, AZ, USA) were cut to the desired length with a Shortix CE capillary column cutter (Scientific Glass Technology Singapore Pte Ltd., Singapore). The capillary cassette was held at a constant temperature of 20 °C. All CE/(MC-)ICP-MS measurements were performed in negative polarity mode (i.e., cathode at the inlet side) using capillaries coated with a successive multiple ionic polymer layers (SMIL) coating consisting of two layers of polybrene (PB) separated by a layer of dextran sulfate (DS). Detailed descriptions of the coating procedures can be found in a previous work of the authors [31] and in section S1 of the Electronic Supplementary Material (ESM).

For sample introduction in bulk MC-ICP-MS analysis, an Aridus II membrane desolvation system (Teledyne CETAC Technologies, Omaha, NE, USA) was used for sample introduction into the Neptune instrument, thus creating dry plasma conditions.

All measurements with the Neptune Plus and the Element 2 instruments were conducted at either medium (MR) or high (HR) mass resolution. The latter condition was selected only because, as a result of extensive wear at the end of the project, the medium-resolution slit could no longer provide the peak separation needed. The MR setting normally suffices to reliably resolve the signals of the sulfur ions and those of the oxygen-based interferences. Because of the low abundance of ^33^S and the isobaric overlap of the signals of ^36^Ar and ^36^S, which would require a higher mass resolution than currently achievable (>10,000), we focused on ^32^S and ^34^S only.

### Reagents and materials

Ultrapure water (resistivity ≥18.2 MΩcm) was obtained from a Milli-Q Element water purification system (Merck Millipore, Molsheim, France). Concentrated trace metal analysis-grade nitric acid (HNO_3_) and hydrochloric acid (HCl) (both Primar Plus from Fisher Chemicals, Leicestershire, UK) were further purified by sub-boiling distillation (twice) with a DST 4000 sub-boiling unit (Savillex, Eden Prairie, MN, USA). Ammonium acetate (99.999%, trace metal basis), methanol (hypergrade), 2-propanol (electronic grade, 99.999%, trace metal basis) and 2-pyridinesulfonic acid (PSA, 97%) were purchased from Sigma-Aldrich (Munich, Germany). The coating agents hexadimethrine bromide (≥95%) and dextran sulfate sodium salt (MW = 40,000 g/mol) were also obtained from Sigma-Aldrich (Munich, Germany). Sodium hydroxide (NaOH, 30%, Suprapur) was purchased from Merck (Darmstadt, Germany). The sulfur standard (ICP-S, 1000 mg/L as (NH_4_)_2_SO_4_ in water) and sodium standard (Na, 10,000 mg/L) were obtained from Chem-Lab NV (Zedelgem, Belgium). Sulfamic acid (≥99.3%) and ammonia solution (20%, Rotipuran Ultra-quality) were purchased from Carl Roth (Karlsruhe, Germany). For comparison of different data evaluation methods and sample-standard bracketing, the IRMs IAEA-S-1 (Ag_2_S; δ^34^S = −0.30‰), IAEA-S-2 (Ag_2_S; δ^34^S = 22.7‰ ± 0.2‰ SD) and IAEA-S-3 (Ag_2_S; δ^34^S = −32.3‰ ± 0.2‰ SD) from the International Atomic Energy Agency (Vienna, Austria) were used. River water samples were collected in August 2019 from the Rhine (Koblenz, Germany, sample Rhi), Scheldt (Ghent, Belgium, Sch) and Lys rivers (Ghent, Belgium, Lys), and from Teltow Canal (Tel), Dahme (Dah), Müggelspree (Mu1) and a small branch canal of Müggelspree (Mu2) in Berlin (Germany). All samples were filtered (0.45 μm) and stored at 4 °C until analysis. The coordinates of the sampling sites can be found in the ESM, Table S1.

### Procedures

For CE/MC-ICP-MS measurements, a 40 mmol/L ammonium acetate background electrolyte (BGE) adjusted to pH 9.7 and a sheath liquid (SL), consisting of 0.01% (by mass) ammonia solution and 10% (by volume) 2-propanol in ultrapure water, were prepared.

The IRMs were digested with concentrated HNO_3_ and HCl following a procedure modified from Das et al. [32], which is described in detail in section S2 of the ESM.

For use in bulk MC-ICP-MS analysis, analyte/matrix separation of sulfate from the IRM solutions, ICP-S and river water samples was accomplished by anion exchange chromatography using AG1-X8 resin (200–400 mesh, Bio-Rad, Watford, UK) following a procedure modified from Han et al. [33], which is described in detail in section S2 of the ESM. All solutions for MC-ICP-MS analysis were diluted to a final sulfur concentration of 600 μg/L in 0.3 mol/L HNO_3_, and 1.2 mg/L Na was added to improve transmission of S through the membrane desolvation system [25]. A solution of PSA in ultrapure water was analyzed via bulk MC-ICP-MS without preceding analyte/matrix separation, because for a pure solution no separation is required [31].

For IRM solutions, ICP-S, PSA solution and river water intended for CE/MC-ICP-MS analysis, no preceding anion exchange chromatography was performed. Instead, analyte/matrix and species separation were accomplished by CE. However, the acidity of the IRM solutions would lead to a deterioration of the electrophoretic separation. Therefore, the acid in IRM solutions was removed by evaporating the IRM digest to dryness at 70 °C and taking up the residue in 5 mL of ultrapure water. This was repeated, and finally the solution was evaporated to dryness once more and the residue taken up in 1 mL of ultrapure water to obtain the standard stock solutions.

The MC-ICP-MS instrument was operated at medium or high mass resolution, and instrument settings were optimized daily while introducing a 20 mg/L sulfur solution prepared from ICP-S through the CE capillary with an internal pressure of 100 mbar. Faraday multiplier gain calibration and baseline determination were conducted each measurement day. Details regarding BGE, SL and IRM preparation can be found in sections S1 and S2 of the ESM.

### Bulk MC-ICP-MS analysis

For evaluating the results obtained with the CE/MC-ICP-MS method, bulk MC-ICP-MS measurements were carried out after preceding off-line sulfur isolation by anion exchange chromatography (ESM, section S2) for the IRM solutions, ICP-S and river water samples. The river water samples were analyzed (including chromatographic isolation) in duplicate. For PSA, no analyte/matrix separation was performed as described above.

At the start of each measurement day, the S isotopic composition of ICP-S to be used as a bracketing standard was calibrated against S-1, S-2 and S-3. ICP-S was then used throughout the session as a bracketing standard for all the samples to correct for instrument drift and the mass bias caused by instrumental mass discrimination. A 0.3 mol/L HNO_3_ blank with added Na was measured at the beginning and end of the analytical session to correct for reagent contamination. The blank level accounted for ≤1% of the measurement signal (BEC = 5–10 μg/L, calculated according to Hanousek et al. [34]), and the on-peak blank intensities were subtracted from the total ion signal intensities measured at the mass-to-charge ratios for the respective isotopes during data evaluation. Between every two measurements, a wash solution (0.3 mol/L HNO_3_) was introduced for 2.5 min until the S signal returned to the blank level, thus eliminating possible carry-over effects. Each sample duplicate was measured three times (i.e., six measurements per sample).

### Sample preparation for species-specific sulfate isotopic analysis

Matching the concentration of standards and samples is important for accurate mass bias correction using an external standard in MC-ICP-MS and is therefore also expected to be relevant in on-line approaches. Thus, the sulfate concentration of the river water samples was determined by CE/ICP-SFMS analysis using the Element 2. Because of the species-unspecific response of ICP-MS (shown, e.g., in a previous work [31]), a different sulfur-containing compound, sulfamic acid, could be used as external calibrant. This enabled the preparation of matrix-matched calibration solutions by dissolving sulfamic acid in river water of the sample Tel, diluted 1:5 with ultrapure water. Calibration solutions were prepared in ten different concentrations ranging from 1.6 to 16 mg/L of sulfur. The Rhine sample was diluted 1:2 and all other samples 1:5 with ultrapure water. The sulfate quantification revealed concentrations ranging from 16 to 68 mg/L of sulfur (see Table S1 in the ESM for detailed results).

**Fig. 1:**
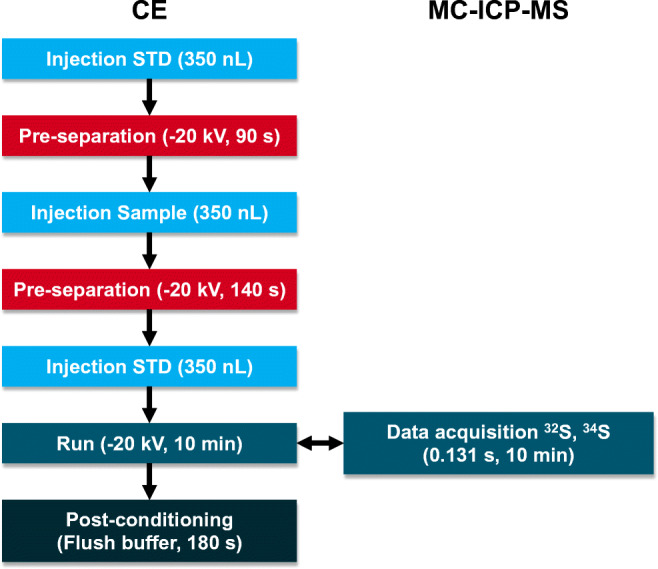
Multiple-injection method for mass bias correction by sample-standard bracketing in CE/MC-ICP-MS. Injection volume calculated with zeecalc [38]

To limit S-1 consumption, the samples and the bracketing standard were diluted to 14 mg/L with ultrapure water prior to CE/MC-ICP-MS measurements, thus only requiring one dilution of the bracketing standard for all measurements. To test the applicability to samples with multiple species, a mixed sample was prepared by spiking the Rhine river water with PSA. For comparability with earlier measurements of single species (sulfate only), samples of Rhine water with sulfate and PSA were diluted such that 14 mg/L of sulfate was present, next to 20 mg/L of PSA. The injections of the bracketing standard were performed in the same way as for the single-species samples (see Fig. 1), ensuring that the peaks of the bracketing standard were appearing before the sample sulfate peak and after the PSA peak. Each sample was measured three times.

### Data evaluation for species-specific sulfate isotopic analysis

For data evaluation, three different methods for calculation of the δ-values from the raw peak signals were compared. The point-by-point (PBP) method uses an average of the raw isotope ratios of every single data point throughout the electrophoretic peak. For the peak area integration (PAI) method, the electrophoretic peak areas of both isotopes are first calculated and then ratioed. Both these methods require a background correction, which is done for each isotope by averaging the background before and after the peak and subtracting this average from the data points within the peak. Because the electrophoretic peaks are not completely symmetrical and different species may have differing peak shapes, 100% of the peak was used for calculating the raw isotope ratios by PAI and PBP. The linear regression slope (LRS) method can be used without background correction. It is based on a two-isotope plot of all data points starting with the background before the electrophoretic peak and ending with the background after the peak. The raw isotope ratio is then represented by the slope of the best-fitting straight line (linear regression) through all data points. All three calculation methods are explained in detail in the literature [2], and a more detailed description of the calculations can be found in section S3 of the ESM.

The δ^34^S value can be calculated directly from the raw isotope ratio results using the following equation (modified from Clough et al. [35]):

$$ {\updelta}^{34}{\mathrm{S}}_{\mathrm{sample}}=\left[\frac{{\left({}^{34}\mathrm{S}{/}^{32}\mathrm{S}\right)}_{\mathrm{sample}}}{{\left({}^{34}\mathrm{S}{/}^{32}\mathrm{S}\right)}_{\mathrm{IAEA}-\mathrm{S}-1}}\times \left(\frac{{\left({\updelta}^{34}\mathrm{S}\right)}_{\mathrm{IAEA}-\mathrm{S}-1}}{1000}+1\right)-1\right]\times 1000 $$

with (^34^S/^32^S)_IAEA-S-1_ as the average of the raw isotope ratios of the corresponding electrophoretic peaks for the standard and normalization to the VCDT scale by including the δ^34^S of IAEA-S-1, which is an assigned value bearing no uncertainty by definition [36].

It has been reported that different amplifier time constants of the Faraday cup detectors used can induce isotope ratio drift during the acquisition of transient signals, which can be corrected for by mathematical methods [37]. In our case, the Neptune Plus instrument used was able to carry out an automatic time-lag correction (*τ*-correction). However, no differences in the resulting δ^34^S values were found irrespective of whether this *τ*-correction was used, but an isotope ratio drift was present in all signals. This could be because the instrumental *τ*-correction may only be suitable for continuous sample introduction and not for transient signals, or that the different isotopes show a slight fractionation within the CE capillary. This will lead to biased results in the PBP and LRS method but not in the PAI method, provided that 100% of the electrophoretic peak area is used for the calculation.

Whereas internal precision can be calculated using the LRS or PBP method, this is not possible for the PAI method. Here, we determined repeatability based on three repeated measurements as twice the standard deviation (2SD). Repeatability can be seen as the major uncertainty component because other factors, e.g., isotope fractionation during separation and measurement, are corrected for by the multiple-injection sample-standard bracketing approach described in the following section. For better comparability, this was also done for the LRS and PBP methods, as well as for bulk MC-ICP-MS analysis. Bulk MC-ICP-MS measurements of the IRMs and ICP-S were performed over the span of 1 year. The median repeatability (2SD) for three consecutive measurements conducted in eight sessions was used for the comparison of IRM results with those obtained using the on-line CE/MC-ICP-MS method. The inter-day repeatability of the CE/MC-ICP-MS was evaluated by measuring ICP-S at least twice each day for 7 days (five times on day 1 and three times on day 6). The inter-day repeatability was calculated as twice the standard deviation (2SD) of these measurements and was compared to inter-day repeatability (2SD) of the bulk MC-ICP-MS measurements of ICP-S performed over 1 year (*n* = 68).

## Results and discussion

### Mass bias correction by external correction

For the introduction of the bracketing standard measured in a sample-standard bracketing sequence, a setup similar to that reported by Martelat et al. [30] using a second nebulizer for standard introduction was considered. However, the addition of the standard aerosol resulted in wet plasma conditions decreasing the width of the ^32^S plateau region because of a significant increase of the intensity of the interfering ^16^O_2_^+^ signal. Also, the use of a desolvation nebulizer was considered. However, systems like the Aridus, employed for bulk MC-ICP-MS measurements of sulfur, require high flow rates of gases and the aspirated liquid for reliable aerosol generation, which is incompatible with the CE/ICP-MS interface.

**Fig. 2:**
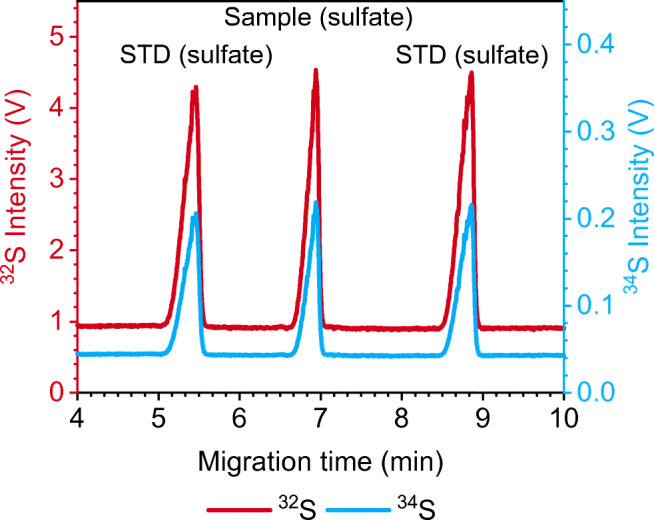
CE/MC-ICP-MS electropherogram of a species-specific on-line isotopic analysis of river water sulfate using the multiple-injection method. Digested IAEA-S-1 reference material was used as bracketing standard (STD) and also contains sulfur in the form of sulfate. The measurement was conducted at MR

Hence, post-separation addition of a liquid standard was no longer considered for sulfur isotopic analysis. Instead, a multiple-injection method (Fig. 1) was developed, thus avoiding manipulation of the CE/ICP-MS interface. The standard is injected twice, before and after the sample, and subsequently the sulfate it contains is separated in the same run as that from the sample, so that one run contains all peaks — of standard and sample — required for obtaining a mass bias-corrected isotope ratio result. Between the different injections, pre-separation steps (i.e., applying a separation voltage for a certain time window) are conducted to ensure complete separation of all peaks (Fig. 2). The duration of the pre-separation steps is dependent on the mobility of standard and sample species and has to be adjusted if other species are measured. Furthermore, separation of analyte and standard species from plugs of the electro-osmotic flow (EOF) (i.e., neutral compounds migrating with the EOF) of preceding injections is necessary. Co-migration with the EOF plug leads to a deteriorated peak shape because the migrating ions will be defocused when entering the plug and refocused when leaving it. The S species is the same in standard and river water samples, and thus method development is not complicated by the aforementioned effects. However, it was also possible to analyze samples containing both sulfate and PSA using this multiple-injection method (as shown in a later section).

This method has the advantage that standard and sample are subjected to the same separation and data evaluation conditions, which is very important for adequate mass bias correction. Using a post-column added standard for external correction brings about that the standard is not subjected to the same separation conditions and is therefore not the preferred approach.

### Evaluation of the isotope ratio calculation methods

For validation of the on-line CE/MC-ICP-MS method developed, three IRMs (IAEA-S-1, IAEA-S-2, IAEA-S-3) were analyzed using one of them, IAEA-S-1, as the bracketing standard. The results obtained by using three different data evaluation methods, PAI, LRS and PBP, are compared in Table 1. It can be seen that the results for S-1 are very promising with the 2SD range including the reference value. The 2SD ranges of the PAI and LRS data are also comparable to those of the off-line results. However, the results for S-2 and S-3 are outside the range of the reference value, thereby showing a systematic bias, and/or have a large 2SD range. Apparently, the large difference between the δ-values of S-2 and S-3 compared to S-1 is a problem for the CE/MC-ICP-MS method. The off-line analysis of S-2 also shows a slightly lower δ^34^S value than the corresponding reference value. This deviation was also reported in another work [39]. Possibly, the underlying effects are higher with the hyphenated technique. Because it was not clear in which range around the δ^34^S value of S-1 there would be no systematic bias, off-line MC-ICP-MS measurements were also conducted for all other samples, so that a verification of the results obtained by on-line CE/MC-ICP-MS was possible. Also, this enabled further investigation on the differences between the PAI and LRS method because a decision as to which method would be better suited for CE/MC-ICP-MS data evaluation was not possible based only on the results of Table 1. The PBP method shows a large 2SD range for all three IRMs and was therefore omitted in later measurements.

**Table 1:** Comparison of data evaluation methods for CE/MC-ICP-MS using different IRMs bracketed with IAEA-S-1 standard. For the analysis of S-1, solutions from IRM aliquots that were digested in parallel were used as sample and bracketing standard. The calculation of δ-values was done based on three different methods, LRS, PAI and PBP. Results for on-line CE/MC-ICP-MS analysis are compared with those obtained via off-line bulk MC-ICP-MS analysis (both at MR) and reference values. For experimental results, twice the standard deviation (2SD) of *n* = 3 consecutive measurements is stated. The certified uncertainty of the reference value of S-2 and S-3 is the standard deviation

Sample	δ^34^S (‰, VCDT)				
	PAI	LRS	PBP	Reference value	Off-line MC-ICP-MS
IAEA-S-1	−0.16 ± 0.25	−0.41 ± 0.32	−0.60 ± 1.72	−0.30^*a*^	−0.30 ± 0.20
IAEA-S-2	21.24 ± 0.23	21.73 ± 0.30	21.16 ± 4.33	22.7 ± 0.2^*a*^	22.26 ± 0.18
IAEA-S-3	−32.20 ± 1.90	−32.32 ± 2.00	−31.96 ± 0.79	−32.3 ± 0.2^*a*^	−32.55 ± 0.15

^*a*^ Coplen and Krouse 1998 [36]

**Fig. 3:**
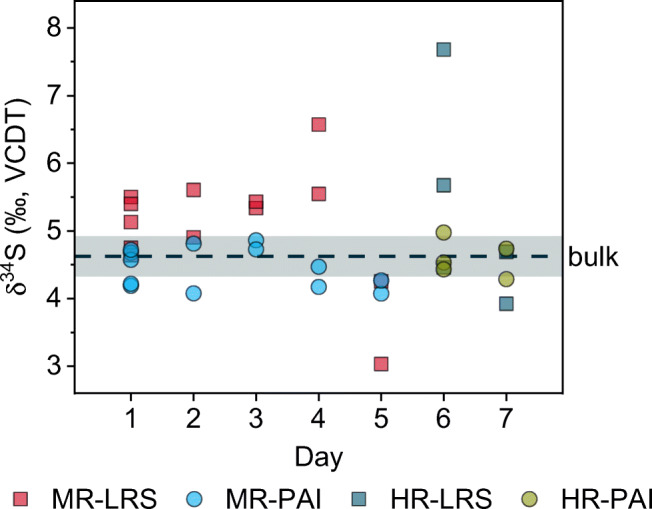
Inter-day repeatability of the isotopic analysis of the ICP-S standard by on-line CE/MC-ICP-MS and bulk MC-ICP-MS. CE/MC-ICP-MS measurements were conducted using two different mass resolution settings, medium resolution (MR) and high resolution (HR), on seven different days. The resulting data were evaluated using two different methods, the LRS (squares) and the PAI (circles) approach. Data points represent single measurements. The gray area represents twice the repeatability standard deviation (±2SD) of the mean (dashed line) of *n* = 68 bulk MC-ICP-MS measurements (MR) conducted over a period of 1 year

By measuring ICP-S on seven different days (Fig. 3), the inter-day repeatability of the CE/MC-ICP-MS method was evaluated. HR was used for the last two days because, as a result of wear, the medium-resolution slit could no longer provide the peak separation needed for resolution of S isotopes and oxygen dimers. Figure 3 shows that the results of different days are similar when using the PAI approach. The PAI-based data also fall within the ±2SD range of the results from bulk MC-ICP-MS measurements. Condensing all measurements leads to overall δ^34^S values for ICP-S of 5.14‰ ± 2.02‰ and 4.49‰ ± 0.57‰ (±2SD, *n* = 18) for the LRS and PAI methods, respectively, compared to 4.62‰ ± 0.30‰ (±2SD, *n* = 68) derived from bulk MC-ICP-MS analyses conducted over a period of 1 year. Compared to the results shown in Table 1 displaying the repeatability of consecutive measurements, the inter-day repeatability of results obtained using the LRS method seems to be much worse than those obtained through calculation with the PAI method. In addition, the PAI-based results also agree with the off-line data, as demonstrated by the overlapping average ±2SD range. Together with the fact that the inter-day repeatability of the on-line PAI method is only roughly twice that of the off-line method, it can be expected that the PAI method is the more reliable of the two data evaluation methods for on-line CE/MC-ICP-MS.

### Species-specific isotopic analysis of river water sulfate

The method developed was applied to the isotopic analysis of river water sulfate using the LRS and PAI methods for the calculation of δ-values. As a proof-of-concept application, it was evaluated whether the repeatability of the method is good enough to reveal differences in the sulfur isotope ratio between different river systems, and whether the data evaluation methods also generate correct δ^34^S values for sulfate in samples with a real matrix. For comparison, bulk MC-ICP-MS measurements of river sulfate isolated by off-line anion exchange chromatography were also conducted. For CE/MC-ICP-MS, S-1 was used as the bracketing standard instead of a secondary standard. Because of the low injection volume of about 347 nL, the standard consumption is very low. For the analysis of one sample with three replicates, the mass of S-1 standard used is 225 ng, whereas for bulk MC-ICP-MS using the Aridus, a total of 5.6 μg would be required. Therefore, ICP-S was used as a secondary in-house standard for bracketing in bulk analysis.

**Fig. 4:**
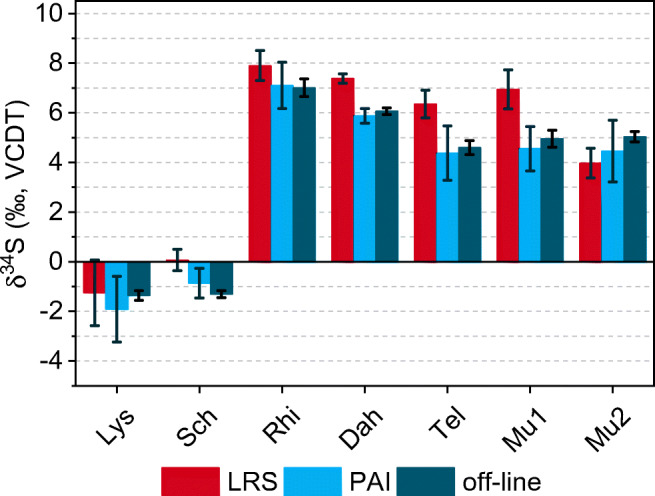
δ^34^S values for river water sulfate obtained via CE/MC-ICP-MS and bulk MC-ICP-MS after prior off-line separation of sulfur by anion exchange chromatography. Error bars represent ±2SD of *n* = 3 consecutive measurements (n = 6 for off-line measurements). All measurements were conducted at MR

It can be shown that while the PAI and the LRS calculation methods give different results for some samples (Sch, Dah, Tel, Mu1), the results from off-line measurements agree with the PAI method (Fig. 4). Thus, the PAI method was considered to give more reliable results. The 2SD range of on-line measurements is obviously somewhat larger than that of off-line measurements, which is due to the transient nature of the signal in the former approach compared to the continuous high-intensity signal in the latter. The on-line approach suffers from the fact that most of the data points within a peak are located on the flanks and have a lower signal intensity than the data point at the peak maximum, especially if 100% of the peak area is used. Nevertheless, the 2SDs of the δ^34^S values of river water sulfate determined by on-line CE/MC-ICP-MS were between 0.3‰ and 1.3‰ only and seem to enable a differentiation between river systems on the basis of sulfate-δ^34^S. The Belgian samples Lys and Sch have very low δ^34^S values standing out from all other samples, and there also seems to be a difference between the Rhine and three of the samples from Berlin (Tel, Mu1, Mu2). For real statistical significance, however, more data would have to be acquired.

### Species-specific isotopic analysis of samples containing multiple species

**Fig. 5:**
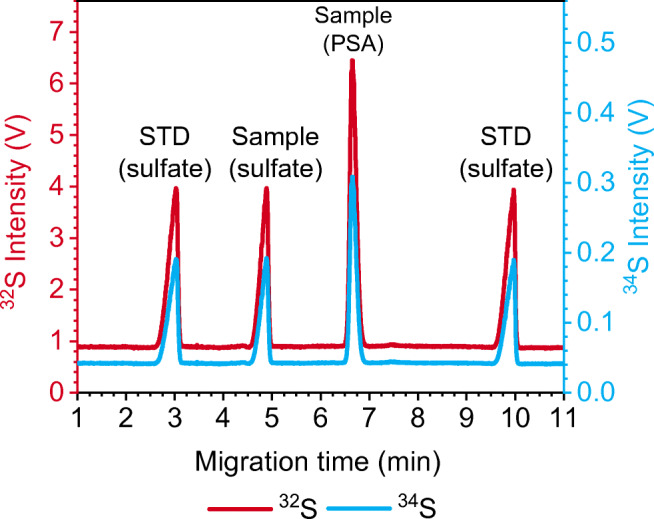
CE/MC-ICP-MS electropherogram allowing for species-specific on-line S isotopic analysis of a sample containing PSA spiked in water from the Rhine River as well as originally present sulfate using the multiple-injection method. The standard (STD) contains sulfur in the form of sulfate. The measurement was conducted at MR

As a perspective for future development, it was also investigated whether the CE/MC-ICP-MS method developed can be used to determine the S isotope ratio in different co-present species. For this experiment, a river water sample from the Rhine River was spiked with PSA, and δ^34^S values were determined for both species. Regarding the multiple-injection sample-standard bracketing, the ideal solution would be to use a third standard peak between the two analytes. However, this approach would become increasingly difficult if more species (>2) were present in the sample because the time window between two analyte peaks might be too small. Thus, the peaks of the bracketing standard were placed before the first and after the last sample species peak (see Fig. 5).

When comparing results derived from the respective single-analyte samples and the mixed sample, good agreement was found for Rhine sulfate (Table 2). For PSA, the δ^34^S value of the mixed sample is somewhat lower than that of the single-analyte sample. Probably, the different peak heights of standard and PSA may be an explanation for this bias. In addition, the 2SD of the bulk MC-ICP-MS measurements is only moderately better than that determined with CE/MC-ICP-MS. This first experiment allows one to expect that also isotopic analysis of multiple species could be possible with reasonable repeatability (2SD) using the multiple-injection sample-standard bracketing approach with standard peaks only before and after all sample analytes.

**Table 2:** Species-specific isotopic analysis of river water spiked with PSA by CE/MC-ICP-MS compared with off-line MC-ICP-MS results of single-analyte samples. For evaluation of on-line data, the PAI method was used. The 2SD of *n* = 3 consecutive measurements (*n* = 6 for off-line MC-ICP-MS of sulfate) is stated. All measurements were conducted at MR

Analyte	δ^34^S (‰, VCDT)	
	CE/MC-ICP-MS	MC-ICP-MS
PSA single	4.97 ± 0.43	5.31 ± 0.32
PSA mixed	4.19 ± 0.49	
Sulfate (Rhi) single	7.10 ± 0.93	7.01 ± 0.35
Sulfate (Rhi) mixed	6.92 ± 0.62	

## Conclusions

In this work, a CE/MC-ICP-MS method for on-line species-specific isotopic analysis of sulfur was developed. The repeatability attainable expressed as 2SD ranged from 0.3‰ to 1.3‰, with the better values approaching those achievable with off-line analysis and the worse ones reflecting the difficulties related to the low sensitivity of the Faraday cup detectors of MC-ICP-MS instruments, which is especially unfavorable for peaks. It is also necessary to use high-purity reagents for the preparation of BGE and SL to keep the sulfur background as low as possible. The in-house preparation of low-sulfur ammonium carbonate buffers with CO_2_ gas [40] may be a possible solution to this problem. The measurement precision (expressed as repeatability), was sufficient to reveal differences in the sulfate-δ^34^S value between river systems, which is a promising result regarding more complex future applications. A proper uncertainty estimation should be done in future work applying this method to assess the results in a more detailed way and evaluate its discrimination power. Future work could also focus on the area of the life sciences because the sample size available is typically very small for biological samples. Low sample consumption is a major advantage of CE, where an injection volume in the nanoliter range can be used without problems, and separations can be developed in aqueous BGE. The method developed could be used for separating sulfur-containing biomolecules (e.g., relevant proteins) from one another and subsequent on-line S isotopic analysis. Also, biomolecules containing metals, e.g., metallothioneins, could be future analytes for species-specific isotopic analysis of metals in biological systems.

## Electronic supplementary material

ESM 1 (PDF 193 kb)
