# Correction to: Method development for on-line species-specific sulfur isotopic analysis by means of capillary electrophoresis/multicollector ICP-mass spectrometry

**DOI:** 10.1007/s00216-021-03474-6

**Published:** 2021-06-21

**Authors:** Sebastian Faßbender, Katerina Rodiouchkina, Frank Vanhaecke, Björn Meermann

**Affiliations:** 1 Federal Institute for Materials Research and Testing (BAM), Division 1.1 - Inorganic Trace Analysis, Richard-Willstätter-Str. 11, 12489 Berlin, Germany; 2 Department of Chemistry, Atomic & Mass Spectrometry – A&MS research unit, Ghent University, Campus Sterre, Krijgslaan 281-S12, 9000 Ghent, Belgium

**Correction to: Analytical and Bioanalytical Chemistry (2020) 412:5637–5646**

10.1007/s00216-020-02781-8

The article Method development for on-line species-specific sulfur isotopic analysis by means of capillary electrophoresis/multicollector ICP-mass spectrometry, written by Sebastian Faßbender, Katerina Rodiouchkina, Frank Vanhaecke and Björn Meermann, was originally published Online First without Open Access. After publication in volume 412, issue 23, page 5637–5646 the author decided to opt for Open Choice and to make the article an Open Access publication. Therefore, the copyright of the article has been changed to ©The Author(s) 2021 and the article is forthwith distributed under the terms of the Creative Commons Attribution 4.0 International License, which permits use, sharing, adaptation, distribution and reproduction in any medium or format, as long as you give appropriate credit to the original author(s) and the source, provide a link to the Creative Commons licence, and indicate if changes were made. The images or other third party material in this article are included in the article’s Creative Commons licence, unless indicated otherwise in a credit line to the material. If material is not included in the article’s Creative Commons licence and your intended use is not permitted by statutory regulation or exceeds the permitted use, you will need to obtain permission directly from the copyright holder. To view a copy of this licence, visit http://creativecommons.org/licenses/by/4.0.

