# Supplementary material for: Method development for on-line species-specific sulfur isotopic analysis by means of capillary electrophoresis/multicollector ICP-mass spectrometry
Source: Anal Bioanal Chem. 2020 Jul 1;412(23):5637–46. doi: 10.1007/s00216-020-02781-8 (PMC8236454; doi:10.1007/s00216-020-02781-8)
Supplement: Supplementary file 1 — (PDF 193 kb) [file 216_2020_2781_MOESM1_ESM.pdf]

Analytical and Bioanalytical Chemistry

Electronic Supplementary Material

Method development for on-line species-specific sulfur isotopic analysis by means of capillary electrophoresis/multicollector ICP-mass spectrometry

Sebastian Faßbender, Katerina Rodiouchkina, Frank Vanhaecke, Björn Meermann

Table of contents

S1: Preparation of BGE and SL, Capillary coating and recoating procedures

S2: Preparation of IRMs, ICP-S and river water samples

S3: Data evaluation

Table S1: River water samples

Table S2: Optimized operating conditions for CE/MC-ICP-MS species-specific isotopic analysis

Table S3: Operating conditions for CE/ICP-SFMS quantitative speciation analysis

Table S4: Operating conditions for MC-ICP-MS bulk isotopic analysis

Table S5: Injection protocol for sample-standard bracketing in CE/MC-ICP-MS (river water samples)

Table S6: Injection protocol for sample-standard bracketing in CE/MC-ICP-MS (PSA sample)

Table S7: Injection protocol for sample-standard bracketing in CE/MC-ICP-MS (mixed PSA and sulfate sample)

S1: Preparation of BGE and SL, Capillary coating and recoating procedures

CE background electrolyte (BGE) stock solution was prepared from ammonium acetate (100 mM solution) adjusted to pH 9.7 with ammonia solution. The stock solution was prepared in metal-free 50 mL centrifuge tubes, sealed and stored at 4 °C in a refrigerator. The BGE was prepared freshly every day by diluting the refrigerated stock solution with ultrapure water. The sheath liquid (SL) was prepared freshly every other day by diluting 50 µL ammonia solution (10%, by mass) and 5 mL 2-propanol with ultrapure water to a final volume of 50 mL. The final ammonia concentration was 0.01% (by mass) and the final concentration of 2-propanol was 10% (by volume).

The SMIL (successive multiple ionic polymer layers) coating consisted of one layer of dextran sulfate (DS) sandwiched between two layers of polybrene (PB). The coating procedure was conducted using the CE instrument only and cooling the capillary cassette down to 15 °C because higher temperatures were found to result in weaker performance of the coating. During the flushing steps, a pressure of 1000 mbar was applied at the capillary inlet. To remove impurities and to activate the silanol groups, the capillary was flushed with methanol (10 min), 1 mol/L HCl (10 min), 1 mol/L NaOH (25 min), 0.1 mol/L NaOH (25 min) and ultrapure water (10 min). Subsequently, the first layer of PB was produced flushing an aqueous solution of 5% PB (by mass) through the capillary for 30 minutes. To stabilize the layer, this was followed by a waiting step (15 min) loading vials with BGE as inlet and outlet to avoid clogging. Afterwards, the coating solution was flushed out with ultrapure water (5 min). This was repeated for the DS layer using a solution of 3% DS (by mass) in ultrapure water and for the second PB layer. Finally, the capillary was flushed with BGE (25 min), and the coating was conditioned by applying a high voltage of -30 kV for 20 minutes. After checking for a stable current, the capillary was flushed again with BGE (15 min) and then installed into the CE-MS sprayer. The produced coating was stable for a few days of non-stop measurements, but to get reproducible results, a recoating procedure was conducted before each sequence of measurements. For the recoating, the capillary was successively flushed with 0.1 mol/L NaOH (10 min), BGE (10 min), 1% PB (by mass) (20 min) and again BGE (10 min), followed by a high voltage conditioning at -30 kV (10 min). After another flush with BGE (5 min), the coating was ready for use.

S2: Preparation of IRMs, ICP-S and river water samples

The Ag_2S reference materials (IAEA-S-1, -S-2, -S-3) were digested following a procedure modified from Das *et al*. [1]. Each standard was separately weighed (~10 mg) into a clean PFA vial, 5 mL of concentrated HNO_3 (purified by sub-boiling distillation) was added and the vial was left open on a 70 °C hot plate overnight. When dry, 3 mL of concentrated HNO_3 and 2 mL of 50% (by volume) concentrated HCl (purified by sub-boiling distillation) was added and the solution was taken to dryness on a 70 °C hot plate overnight. The dry residue was taken up in 0.03 mol/L HNO_3 and purified using an anion exchange resin (AG1-X8, 200-400 mesh, Bio-Rad) following a procedure modified from Han *et al.* [2]. Before use, the resin was subjected to a pre-cleaning in bulk with the following procedure, executed twice: leaching with 6 mol/L HCl for a day, decanting the solution, rinsing with ultrapure water. For the anion exchange column purification, 1 mL of the pre-cleaned resin was loaded into 2 mL PP+PE columns (TrisKem International, Bruz, France) and converted to nitrate form by passing through 10 mL of 3 mol/L HNO_3. The resin was conditioned with 20 mL of 0.03 mol/L HNO_3 before 1 mL of sample solution was loaded. Matrix elements were then removed by washing the resin with 10 mL of 0.03 mol/L HNO_3 and S was eluted with 5 mL of 0.3 mol/L HNO_3. The typical recovery after digestion of the IAEA standards was >96% and the recovery after separation was >93%. Purified IAEA solutions could be run directly for the bulk MC-ICP-MS measurements, but for CE/MC-ICP-MS measurements aliquots of these reference materials were dried in clean PFA vials and taken up in ultrapure water before analysis.

The same purification protocol as for the reference materials was applied for the river water samples prior to bulk MC-ICP-MS measurement.

S3: Data evaluation

For data evaluation, three different methods for calculation of the δ-values from the raw peak signals were compared. The point-by-point (PBP) method uses an average of the raw isotope ratios for every single data point throughout the electrophoretic peak:

$$(^{34}S/^{32}S)_{PBP}=\frac{\Sigma(^{34}S/^{32}S)_i}{n}$$

$(^{34}S/^{32}S)_{PBP}$ is the raw isotope ratio of a peak evaluated by the PBP method, $(^{34}S/^{32}S)_i$ the raw isotope ratio for one data point of the peak and n the number of data points constituting the peak.

For the peak area integration (PAI) method, the electrophoretic peak areas are first calculated for both isotopes and then ratioed:

$$(^{34}S/^{32}S)_{PAI}=\frac{\Sigma(^{34}S)_i}{\Sigma(^{32}S)_i}$$

$(^{34}S/^{32}S)_{PAI}$ is the raw isotope ratio of a peak evaluated by the PAI method, and $(^{34}S)_i$ and $(^{32}S)_i$ are the intensities for one data point of the peaks for ^{34}S and ^{32}S, respectively.

Both these methods require a background correction, which is done for each isotope by averaging the background before and after the peak and subtracting this average from the data points within the peak:

$$(^{34}S)_i=(^{34}S)_{i,uncorr}-\frac{\Sigma(^{34}S)_{j,bkg}}{m}$$

$(^{34}S)_i$ is the background-corrected ^{34}S intensity for one data point of a peak, $(^{34}S)_{i,uncorr}$ the uncorrected ^{34}S intensity for one data point of a peak, $(^{34}S)_{j,bkg}$ the ^{34}S intensity for one data point of the background, and m the number of data points considered for the background evaluation.

Because the electrophoretic peaks are not completely symmetrical and different species may have differing peak shapes, the entire peak was used for calculating the raw isotope ratios by PAI and PBP.

The linear regression slope (LRS) method can be used without background correction. It is based on a two-isotope plot of all data points starting with the background before the electrophoretic peak and ending with the background after the peak. The isotope ratio is then represented by the slope of the best-fitting straight line (linear regression) through all data points. The slope was calculated by using the LINEST function of Microsoft Excel software.

The $\delta^{34}S$ value can be calculated directly from the raw isotope ratio results using the following equation:

$$\delta^{34}S_{sample}=\left[\frac{(^{34}S/^{32}S)_{sample}}{(^{34}S/^{32}S)_{IAEA\text{-}S\text{-}1}}\times\left(\frac{(\delta^{34}S)_{IAEA\text{-}S\text{-}1}}{1000}+1\right)-1\right]\times 1000$$

with $(^{34}S/^{32}S)_{IAEA\text{-}S\text{-}1}$ as the average of the raw isotope ratios of the corresponding electrophoretic peaks for the standard and normalization to the VCDT scale by including the $\delta^{34}S$ value of IAEA-S-1, which is an assigned value bearing no uncertainty by definition.

Table S1 River water samples

Sample	Location	River	km	Coordinates		Sulfate concentration (mg/L)
				Latitude	Longitude	
Rhi	Koblenz (GER)	Rhine	590.3	50°20’58.5’’N	7°35’59.5’’E	15.8 ± 0.3
Tel	Berlin (GER)	Teltow Canal	36	52°25’31.5’’N	13°32’30.9’’E	61.2 ± 1.5
Dah	Berlin (GER)	Dahme	34	52°26’38.1’’N	13°34’32.3’’E	67.9 ± 2.5
Mu1	Berlin (GER)	Müggelspree	1	52°26’46.1’’N	13°35’04.8’’E	65.2 ± 0.2
Mu2	Berlin (GER)	Müggelspree branch canal	/	52°26’39.8’’N	13°25’04.9’’E	63.1 ± 0.2
Sch	Ghent (BEL)	Scheldt	/	51°02’43.0’’N	03°44’38.7’’E	35.2 ± 1.4
Lys	Ghent (BEL)	Lys	/	51°02’19.5’’N	03°41’25.0’’E	32.7 ± 1.3

Table S2 Optimized operating conditions for CE/MC-ICP-MS species-specific isotopic analysis

Parameter	Value
MC-ICP-MS parameters	
Instrument	Neptune Plus (Thermo Scientific)
RF power (W)	1200
Sampling cone	Jet (Ni)
Skimmer cone	X-skimmer (Ni)
Resolution	Medium (~4,000) / High (~10,000)
Auxiliary gas flow rate (L/min)	0.8
Sample gas flow rate (L/min)	0.15-0.35
Faraday cup configuration	H1 (^{32}S), H4 (^{34}S)
Data acquisition	0.131 s integration, 1 Block, 1-6000 cycles
CE parameters	
Instrument	Agilent 7100 CE
Capillary	75 µm i.d., 100 cm length, fused silica
Capillary coating	PB-DS-PB SMIL
Background electrolyte (BGE)	40 mmol/L NH_4Ac, adjusted to pH 9.7 ($NH_{3(aq)}$)
Cassette temperature (°C)	15
Injection	50 mbar, 80 s (347 nL)[a]
Voltage (kV)	-20
Internal pressure (mbar)	15
Postconditioning	3 min Flush BGE
CE/MC-ICP-MS interface parameters	
Nebulizer	Burgener Mira Mist CE
Spray chamber	8 mL Quartz, drainless with make-up gas
Nebulizer gas pressure (psi)	85 (external)
Sheath liquid (SL)	0.01% (by mass) NH_4OH, 10% (by volume) 2-propanol in ultrapure water, pH 10
Sheath liquid flow rate (µL/min)	10

[a] injection volume calculated with zeecalc [3].

Table S3 Operating conditions for CE/ICP-SFMS quantitative speciation analysis

Parameter	Value
ICP-SFMS parameters	
Instrument	Element 2 (Thermo Scientific)
RF power (W)	1250
Auxiliary gas flow rate (L/min)	1.15
Additional gas 1 flow rate (L/min)	0.300
Nuclides monitored	^{32}S, ^{34}S
Resolution	Medium (~4,000)
Sample time (s)	0.002
CE parameters	
Instrument	Agilent 7100 CE
Capillary	75 µm i.d., 60 cm length, fused silica
Capillary coating	PB-DS-PB SMIL
Background electrolyte (BGE)	20 mmol/L NH_4Ac, pH 9.7
Cassette temperature (°C)	20
Injection	50 mbar, 20 s (145 nL)[a]
Voltage (kV)	-20
Internal pressure (mbar)	15
Postconditioning	1 min Flush BGE
CE/MC-ICP-MS interface parameters	
Nebulizer	Burgener Mira Mist CE
Spray chamber	8 mL Quartz, total consumption with make-up gas
Nebulizer gas pressure (psi)	85 (external)
Sheath liquid (SL)	0.01% (by mass) NH_4OH, 10% (by volume) 2-propanol in ultrapure water, pH 10
Sheath liquid flow rate (µL/min)	10

[a] injection volume calculated with zeecalc [3].

Table S4 Operating conditions for MC-ICP-MS bulk isotopic analysis

Parameter	Value
MC-ICP-MS parameters	
Instrument	Neptune Plus (Thermo Scientific)
RF power (W)	1200
Sampling cone	Standard (Ni)
Skimmer cone	H-skimmer (Ni)
Resolution	Medium (~4,000)
Auxiliary gas flow rate (L/min)	0.8
Sample gas flow rate (L/min)	0.8-0.9
Faraday cup configuration	H1 (^{32}S), H4 (^{34}S)
Data acquisition	0.262 s integration, 9 Blocks, 5 cycles
Membrane desolvation system parameters	
Desolvator	Aridus II (Cetac Technologies)
Nebulizer	PFA 100 (ESI)
Sweep gas flow rate (L/min)	9-10.5
N_2 additional gas flow rate (mL/min)	0
Sample uptake rate (µg/min)	100
Spray chamber temperature (°C)	110
Desolvator temperature (°C)	160

Table S5 Injection protocol for standard sample bracketing in CE/MC-ICP-MS (river water samples)

No.	Inlet-Vial	Time (s)	Pressure (mbar)	Voltage (kV)
1	H_2O	5	50	
2	Standard	80	50	
3	BGE	5	50	
4	BGE	90	0	-20
5	H_2O	5	50	
6	Sample	80	50	
7	BGE	5	50	
8	BGE	140		-20
9	H_2O	5	50	
10	Standard	100	50	
11	BGE	5	50	

Table S6 Injection protocol for sample-standard bracketing in CE/MC-ICP-MS (PSA sample)

No.	Inlet-Vial	Time (s)	Pressure (mbar)	Voltage (kV)
1	H_2O	5	50	
2	Standard	80	50	
3	BGE	5	50	
4	BGE	60	0	-20
5	H_2O	5	50	
6	Sample	80	50	
7	BGE	5	50	
8	BGE	230		-20
9	H_2O	5	50	
10	Standard	100	50	
11	BGE	5	50	

Table S7 Injection protocol for sample-standard bracketing in CE/MC-ICP-MS (mixed PSA and sulfate sample)

No.	Inlet-Vial	Time (s)	Pressure (mbar)	Voltage (kV)
1	H_2O	5	50	
2	Standard	80	50	
3	BGE	5	50	
4	BGE	120	0	-20
5	H_2O	5	50	
6	Sample	80	50	
7	BGE	5	50	
8	BGE	320		-20
9	H_2O	5	50	
10	Standard	80	50	
11	BGE	5	50	

References

[1] Das A, Chung C-H, You C-F. Disproportionately high rates of sulfide oxidation from mountainous river basins of Taiwan orogeny: Sulfur isotope evidence. Geophys Res Lett. 2012;39:L12404.

[2] Han S-H, Varga Z, Krajkó J, Wallenius M, Song K, Mayer K. Measurement of the sulphur isotope ratio ($^{34}S/^{32}S$) in uranium ore concentrates (yellow cakes) for origin assessment. J Anal At Spectrom. 2013;28:1919-25.

[3] González-Ruiz V, Drouin N, Reginato E, Rudaz S, Schappler J. "zeecalc 1.0", available online at http://www.unige.ch/sciences/pharm/fanal/lcap/.
